# Adiponectin and resistin modulate the progression of Alzheimer´s disease in a metabolic syndrome model

**DOI:** 10.3389/fendo.2023.1237796

**Published:** 2023-09-04

**Authors:** Pedro Cisternas, Camila Gherardelli, Joel Gutierrez, Paulina Salazar, Carolina Mendez-Orellana, G. William Wong, Nibaldo C. Inestrosa

**Affiliations:** ^1^ Instituto de Ciencias de la Salud, Universidad de O’Higgins, Rancagua, Chile; ^2^ Centro de Envejecimiento y Regeneración (CARE-UC), Departamento de Biología Celular y Molecular, Facultad de Ciencias Biológicas, Pontificia Universidad Católica de Chile, Santiago, Chile; ^3^ Carrera de Fonoaudiología, Departamento Ciencias de la Salud, facultad Medicina, Pontificia Universidad Católica de Chile, Santiago, Chile; ^4^ Department of Physiology, The Johns Hopkins University School of Medicine, Baltimore, MD, United States; ^5^ Center for Metabolism and Obesity Research, The Johns Hopkins University School of Medicine, Baltimore, MD, United States; ^6^ Centro de Excelencia en Biomedicina de Magallanes (CEBIMA), Universidad de Magallanes, Punta Arenas, Chile

**Keywords:** adiponectin, resistin, obesity, Alzheimer´s disease, glucose metabolism

## Abstract

Metabolic syndrome (MetS), a cluster of metabolic conditions that include obesity, hyperlipidemia, and insulin resistance, increases the risk of several aging-related brain diseases, including Alzheimer’s disease (AD). However, the underlying mechanism explaining the link between MetS and brain function is poorly understood. Among the possible mediators are several adipose-derived secreted molecules called adipokines, including adiponectin (ApN) and resistin, which have been shown to regulate brain function by modulating several metabolic processes. To investigate the impact of adipokines on MetS, we employed a diet-induced model to induce the various complications associated with MetS. For this purpose, we administered a high-fat diet (HFD) to both WT and APP/PSN1 mice at a pre-symptomatic disease stage. Our data showed that MetS causes a fast decline in cognitive performance and stimulates Aβ_42_ production in the brain. Interestingly, ApN treatment restored glucose metabolism and improved cognitive functions by 50% while decreasing the Aβ_42/40_ ratio by approximately 65%. In contrast, resistin exacerbated Aβ pathology, increased oxidative stress, and strongly reduced glucose metabolism. Together, our data demonstrate that ApN and resistin alterations could further contribute to AD pathology.

## Introduction

Alzheimer’s disease (AD) is the most common age-related neurodegenerative disorder, and it is characterized by a progressive and gradual decline in cognitive functions (1, 2). Neuropathologically, AD is defined by extracellular deposits mainly composed of amyloid-β (Aβ), known as amyloid plaques, and by neurofibrillary tangles containing hyperphosphorylated tau protein (1, 3). Accumulating evidence has also demonstrated reduced cerebral glucose utilization that precedes clinical symptoms and correlates with disease progression (4, 5). The genetic component only explains approximately 5% of all AD cases, however, the vast majority (∼95%) appear sporadic and associated with several susceptibility genes and environmental factors (6, 7). Among the latter, acquired factors, such as diabetes, hypertension, dyslipidemia, and mid-life obesity, whose accumulation is known as metabolic syndrome (MetS), have been shown to increase AD (8–16).

Obesity (BMI ≥ 30 kg/m^2^) is defined as increased body weight due to excessive or abnormal fat accumulation. According to the WHO, over 650 million people worldwide were estimated to be obese in 2016, and its prevalence is expected to rise dramatically, increasing the health costs related to this disorder. For decades, the adipose tissue was considered an energy storage depot with protective properties. However, it has been demonstrated that the adipose tissue represents the body’s largest endocrine gland (17–20). Indeed, this endocrine tissue is responsible for the liberation of hundreds of types of molecules collectively called adipokines, which facilitate adipose tissue crosstalk with other organs, including the brain (21, 22). Among the most studied adipokines in the brain are ApN and resistin, which have been associated with AD progression due to their role in glucose metabolism and effect on insulin resistance (23–26). ApN is the most abundant adipokine, and its secretion is inversely correlated to fat mass, meaning that it decreases in obesity (27, 28). Importantly, ApN has been postulated to act as a neuroprotective molecule against AD progression due to its role in insulin-sensitizing and anti-inflammatory properties (29–32). Contrary to ApN, resistin levels increase in obesity, and it has been proposed as a risk factor for dementia, as it counteracts the effects of insulin and promotes inflammation in obesity (33, 34). Although these adipokines play critical roles in energy homeostasis and stimulate energy expenditure, their effect on AD progression remains unclear (35).

In the present work, we studied the effect of ApN and resistin in wild-type and AD-transgenic obese mouse models. Our results demonstrate that contrary to resistin, ApN administration prevented a decrease in glucose metabolism and induced a partial recovery in cognitive functions. Moreover, ApN treatment led to a substantial decrease in aggregates of the most pathogenic Aβ species, Aβ_42_, whereas resistin resulted in smaller Aβ aggregates size. Interestingly, obesity caused a general metabolic dysfunction and an increase in the expression of oxidative markers, a substantial decrease in cerebral glucose utilization, and a reduction in memory/learning functions. Together, our findings support the view that restoring normal ApN and resistin levels could delay AD onset, and their pharmacological modulation could be used as a tool against AD pathology.

## Materials and methods

### Animals and ethical standards

**Figure 1:**
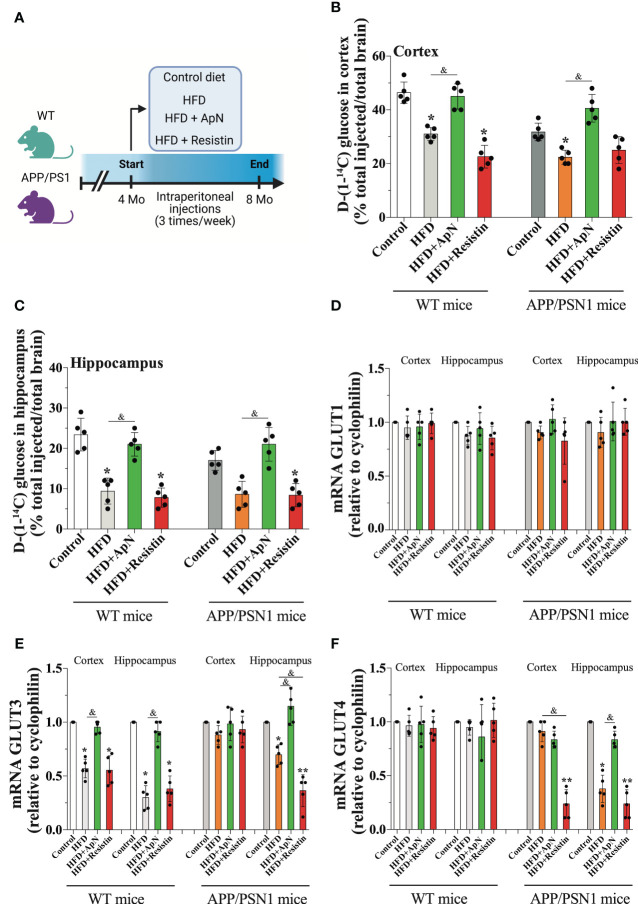
Glucose uptake is induced by ApN. **(A)** Schematic of experimental design. 4 months-old wild-type or APP/PSN1 mice were fed with either a control diet or high-fat diet (HFD), and received intraperitoneal injections of adiponectin (ApN), or resistin 3 times per week for 4 months. **(B, C)** Radioactive glucose was tail‐vein injected and 15 min later glucose uptake was measured in the cortex or hippocampus. **(D-F)** The effects of the HFD and adipokines treatments on the mRNA levels of the glucose transporters Glut1, Glut3, and Glut4 were measured in the cortex and hippocampus using qRT–PCR. Means ± SEM are shown (n = 5 mice per condition in 3 technical replicates). Statistical significance was determined by one-way ANOVA followed by Bonferroni’s multiple comparison posthoc test. *p < 0.05 and **p < 0.01, compared with control group; ^&^p < 0.05, compared with specified group.

As a control group, we used 4-month-old (pre-symptomatic stage) male wild-type (WT) mice (CB57BL/6), distributed with simple randomization to allocate them into different cages. As an AD model, we used male APPswe/PSN1dE9 (#034829-JAX) (4-month-old, APP/PSN1, as an asymptomatic model before starting the treatment). APP/PSN1 animals co-express the Swedish (K594M/N595L) mutation of a chimeric mouse/human APP (Mo/HuAPP695swe) together with the human exon-9-deleted variant of PSN1 (PSN1-dE9); these mice secrete elevated levels of human Aβ peptide (36, 37). We divided both the WT and AD model groups into four subgroups each, with 4-8 animals in each subgroup (**Figure 1A**). In the first subgroup, animals were fed with basal control diet (with 10% energy from fat, #7024, TestDiet), and in the second subgroup, mice were fed a high-fat diet (HFD, with 45% energy from fat, #58G8 TestDiet). Both of these animals’ subgroups were injected with saline solution as a vehicle three times per week for 16 weeks. The third and fourth subgroups were fed with HFD and received an intraperitoneal injection of ApN (#SRP4902, Sigma-Aldrich, 6 mg/kg) or resistin (#SRP4560, Sigma-Aldrich, 1 mg/kg) three times per week (on Monday, Wednesday, and Saturday) for 16 weeks (38). After the treatment, all animals in each group were subjected to cognitive performance tests. Only animals that completed the entire treatment and appeared healthy were used for the studies. For the inclusion/exclusion criterion, we evaluated the weight, and a visual inspection of signs of stress and distress was performed. The animals were obtained from our colony at the Faculty of Biological Sciences, Pontificia Universidad Católica de Chile. Animals were housed in a ventilated room with a natural photoperiod and controlled temperature (yearly minimum = 13.4 ± 0.2°C; yearly maximum = 24.9 ± 0.2°C) and humidity (40-60%). All experiments followed the National Institutes of Health guidelines (NIH, Baltimore). The Bioethical and Biosafety Committee approved all procedures of the Faculty of Biological Sciences of the Pontificia Universidad Católica de Chile. All efforts were made to minimize animals’ distress and suffering, reducing the number of animals used. Measurements of animal weight and food and liquid intake were obtained once per week during the treatment. The order of injection, decapitation, and behavioral testing was randomized

### Biochemical analysis

At the end of the treatment, glucose, cholesterol, insulin, and triglycerides were measured from blood samples obtained from 4 mice per group. Intracardiac blood was collected before decapitation, after 6 h of fasting, and the serum was separated via centrifugation and stored at -20°C for later analysis via immunoassay and chemiluminescence. Glucose levels were measured according to the hexokinase/G-6-PDH method using Architect Analyzer (Abbott Laboratories). Insulin levels were measured via chemiluminescence (Beckman Coulter) and cholesterol levels were enzymatically assessed using an Architect c8000 analyzer. HOMA, an insulin resistance index, was calculated using the following formula: HOMA-R = fasting glucose (mmol/L) × fasting insulin (μU/ml)/22.5. Triglycerides were assessed enzymatically using the Architect c8000 analyzer. ApN and resistin levels were measured using the ApN mouse ELISA Kit (#KMP0041, ThermoFisher) and the mouse Resistin ELISA Kit (#ab205574, Abcam), according to the manufacturer’s instructions.

### Quantification of ApN, resistin and glucose in the cerebrospinal fluid

To assess the changes in the levels of both ApN and resistin in the brain, we measured both adipokines in CSF (n = 5 mice per group). After the cognitive tests, mice were anesthetized by isofluorane administration, and then a 30-G needle attached to a 1 mL syringe was carefully inserted into the dura mater. CSF was aspirated by pulling back the syringe plunger; the CSF obtained was free of blood contamination and was kept at -20 °C for later study (39). To measure ApN and resistin levels, we used the ApN mouse ELISA Kit (#KMP0041, ThermoFisher) and the mouse Resistin ELISA Kit (#ab205574, Abcam), according to manufacturer’s instructions (40, 41). Glucose in CSF was measured using the hexokinase/G-6-PDH method as in blood.

### D-[1-14C] glucose biodistribution

Upon completing cognitive tests, 5 mice from each group were injected with D-[1-^14^C] glucose (#NEC043, PerkinElmer) via the tail vein. Briefly, mice were anesthetized with isoflurane and injected intravenously via the tail with 50 μCi of tracer diluted to a final volume of 20 μL in isotonic saline. Following a 15 min uptake period, the animals were euthanized and tissues were collected. Tissue radioactivity was quantified by liquid scintillation. D-[1-^14^C] glucose levels were normalized to the weight of resected tissue and expressed as the percent of injected dose (42, 43).

### Quantitative real-time PCR

After drug treatment, mRNA was obtained from cortical or hippocampal tissue and used to generate cDNA. Quantitative real-time RT–PCR (qRT–PCR) was conducted using SYBR master mix (#4368577, ThermoFisher Scientific), with the program recommended by the manufacturer and as published previously (38). As a reference, we used the housekeeping gene cyclophilin (Ppib), and the relative Ct values of each gene were calculated using the delta Ct, in comparison with the control gene. Duplicated control reactions for every sample without reverse transcription were included to ensure that PCR products were not due to the amplification of contaminated genomic DNA. We used the following sets of primers: cyclophilin F (5’-TGGAGATGAATCTGTAGGAGGAG-3’) and R (5’- TACCACATCCATGCCCTCTAGAA-3), Glut1 (Slc2a1) F (5’-ATGGATCCCAGCAGCA AGAAG-3’) and R (5’-AGAGACCAAAGCGTGGTGAG-3’), Glut3 (Slc2a3) F (5’-GGATCCCTTGTCCTTCTGCTT-3’) and R (5’-ACCAGTTCCCAATGCACACA-3’), Glut4 (Slc2a4) F (5’-CGGCTCTGACGATGGGGAA-3’) and R (5’-TTGTGGGATGGAA TCCGGTCCCGATA-3’). All primers were purchased from IDT Integrated DNA Technologies.

### Immunofluorescence

Immunofluorescence was performed in brain slices as previously described (44). Briefly, 40 μm hippocampal slices (Bregma -1.85 to -1.7 mm) were washed three times in ice-cold phosphate-buffered saline (PBS), permeabilized for 30 min with 0.2% Triton X-100 in PBS, and washed with ice-cold PBS. The slices were subsequently incubated in blocking solution (0.2% bovine serum albumin, in PBS) for 1 h at room temperature, followed by overnight incubation at 4°C with primary antibodies; anti-GFAP (#Z0334, Dako) and anti-4-HNE (#ab46545, Abcam). After incubation, the slices were extensively washed with PBS and subsequently incubated with Alexa-conjugated secondary antibodies (Molecular Probes) for 2 h at 37°C. Nuclear staining was performed by treating the slices with Hoechst. The slices were subsequently mounted and analyzed by fluorescence microscopy. Stained brain sections were photographed using a Zeiss LSM 5 Pascal confocal microscope. Images were then loaded onto ImageJ for analysis. The areas for measurement were selected by performing a manual threshold adjustment or direct manual selection of regions of interest in heterogeneously stained sections.

### Glucose uptake analysis

Hippocampal slices were prepared according to standard procedures. Briefly, we sectioned transverse slices (350 μm) from the dorsal hippocampus in cold artificial cerebrospinal fluid (ACSF; 124 mM NaCl, 26 mM NaHCO_3_, 3 mM D- glucose, 2.69 mM KCl, 1.25 mM KH_2_PO_4_, 2.5 mM CaCL_2_, 1.3 mM MgSO_4_, and 2.60 mM NaHPO_4_) using a vibratome (LeicaVT 1000s). Slices were incubated in ACSF for 1 h at room temperature, then were washed with washing buffer (15 mM HEPES [#H3375, Sigma Millipore], 135 mM NaCl [#s3014, Sigma-Aldrich], 5 mM KCl [#P5405 Sigma-Aldrich], 1.8 mM CaCl_2_ (#C1016 Sigma-Aldrich), and 0.8 mM MgCl_2_ [#208337, Sigma-Aldrich]) supplemented with 0.5 mM glucose. Then, the slices were incubated for 30 min with 1-1.2 μCi 2-[1,2-^3^H(N)]-deoxy-D-glucose (#NET328250UC, PerkinElmer) at a final specific activity of 1-3 disintegrations/min/pmol (~1 mCi/mmol). Glucose uptake was arrested by washing the cells with ice-cold PBS supplemented with 1 mM HgCl_2_ (#203777, Sigma-Aldrich). The incorporated radioactivity was quantified by liquid scintillation counting (45).

### Determination of the glycolytic rate

Glycolytic rates were determined as previously described (45). Briefly, hippocampal slices were placed in tubes containing 5 mM glucose and then washed twice in Krebs–Henseleit solution (11 mM Na_2_HPO_4_, 122 mM NaCl, 3.1 mM KCl, 0.4 mM KH_2_PO_4_, 1.2 mM MgSO_4_, and 1.3 mM CaCl_2_, pH 7.4) containing the appropriate concentration of glucose. After equilibration in 0.5 mL of Hank’s balanced salt solution/glucose (#14025076, Thermo Fisher) at 37°C for 30 min, 0.5 mL of Hank’s balanced salt solution containing various concentrations of [3-^3^H] glucose (#NET331, Perkin-Elmer) was added, with a final specific activity of 1-3 disintegrations/min/pmol (~1 mCi/mmol). Aliquots of 100 μL were then transferred to another tube, placed inside a capped scintillation vial containing 0.5 mL of water, and incubated at 45°C for 48 h. After this vapor-phase equilibration step, the tube was removed from the vial, the scintillation mixture was added, and the ^3^H_2_O content was determined by counting over a 5-min period.

### Quantification of ADP and ATP levels

Brain tissues or hippocampal slices were treated with described activators/inhibitors, and ATP levels were measured using an ATP determination kit (#A22066, Invitrogen/Molecular Probes) (46). ADP levels in slices were measured using an ADP Assay Kit (#ab83359, Abcam), according to the manufacturer’s instructions (47).

### Pentose phosphate pathway measurement

Glucose oxidation via the PPP was measured as previously described based on the difference in ^14^CO_2_ production from [1-^14^C] glucose (decarboxylated in the 6-phosphogluconate dehydrogenase-catalyzed reaction and in the Krebs cycle) and [6-^14^C] glucose (only decarboxylated in the Krebs cycle) (48). Hippocampal slices were washed with ice-cold PBS and collected, then slices were kept in O_2_-saturated Krebs Henseleit buffer (11 mM Na_2_HPO_4_, 122 mM NaCl, 3.1 mM KCl, 0.4 mM KH_2_PO_4_, 1.2 mM MgSO_4_, and 1.3 mM CaCl_2_, pH 7.4), and this suspension was placed in Erlenmeyer flasks with another 0.5 mL of the Krebs Henseleit solution containing 0.5 μCi D-[1-^14^C] glucose or 2 μCi D-[6-^14^C] glucose and 5.5 mM D-glucose (final concentration). The Erlenmeyer flasks were equipped with a central well containing an Eppendorf tube with 500 μL of benzethonium hydroxide. The flasks were flushed with O_2_ for 20 s, sealed with rubber caps, and incubated for 60 min in a 37°C water bath with shaking. The incubations were stopped by the injection of 0.2 mL of 1.75 M HClO_4_ into the main well, although shaking was continued for another 20 min to facilitate the trapping of ^14^CO_2_ by benzethonium hydroxide. Radioactivity was assayed by liquid scintillation spectrometry. Both D-[1-^14^C]glucose (#NEC043) and D-[1-^16^C]glucose (#NEC045) were purchased from Perkin-Elmer.

### Large open-field test

A 120 × 120 cm transparent Plexiglas arena with 35 cm-high transparent walls was used to study locomotor and stress behavior in our mouse model. The open field, which measured 40 × 40 cm, was defined as the field’s center area. Data were collected using an automatic tracking system (HVS Imagen). Each mouse was placed alone in the center of the open field, and its behavior was tracked for 20 min, as described previously (49). At the end of the session, the mouse was returned to its home cage. The parameters measured included total time moving and the number of times the mouse crossed the center area of the arena (50). All behavioral tests were performed by the same person at least 3 days after treatment.

### Novel object recognition and novel object localization

The NOR and NOL tasks were performed as previously described (51). Mice were habituated to the experimental room in the experimental cages for 3 consecutive days for 30 min per day (3 consecutive days) and 1 h on the testing day. The task occurred in a 120×120 cm transparent Plexiglas arena with 35 cm-high transparent walls containing two identical objects placed at specific locations. For object familiarization, mice were allowed to explore the arena for 10 min. The animals were subsequently returned to their home cages for 1 h, followed by a 5-min exposure to a novel localization of one of the familiar objects (NOL). The mice were again returned to their home cages for 1 h and were subsequently exposed to a novel object (NOR) for 5 min. The mice had no observed baseline preference for the different objects. An object preference index was determined by calculating the time spent near the relocated/novel object divided by the cumulative time spent with both the familiar and relocated/novel objects. The cages were routinely cleaned with ethanol following mouse testing/habituation of the mice.

### Determination of Aβ peptide levels

Two sandwich ELISAs specific for Aβ_40_ (#EZBRAIN40, EMD Millipore) and Aβ_42_ (#EZBRAIN42, EMD Millipore) were employed to determine the concentrations of Aβ peptides. Briefly, hippocampal and cortical homogenates from each animal were diluted to 1 μg/μl in a homogenization buffer containing protease and phosphatase inhibitors. Protein homogenates (100 μl) were prepared and measured, according to the manufacturer’s instructions.

### Thioflavin S staining for amyloid plaques

Thioflavin S (ThS, #t1892, Millipore Sigma) staining was performed on 40 μm sections mounted on gelatin-coated slides. After dehydration and rehydration in ethanol and xylene, slides were incubated in distilled water for 10 min and then immersed in ThS solution (0.1% ThS in 70% ethanol) for 5 min. The slides were then washed twice with 70% ethanol for 30 sec. The sections were covered with a coverslip and antifade mounting medium in the dark. Five hippocampal or cortical sections were analyzed per animal. To quantify the Aβ plaque area, we used the freehand selection tool in Image J by manually marking the perimeter of each plaque.

### Statistical analysis

All Statistics were performed using the software Prism9 version 9.1.1. (GraphPad Prism). Sample size is reported in figure legends and represents independent samples used in the analysis. The results are expressed as means ± SEM. Data were analyzed by one-way or two-way analysis of variance (ANOVA), followed by Bonferroni’s *post hoc* test; *p < 0.05, **p < 0.01, and ***p < 0.001 were considered significant differences. To test the presence of outliers, we used the Prism software, however, no outliers were detected.

## Results

### HFD induces MetS in WT and APP/PSN1 animals

To study the effect of adipokines on MetS, we used a diet-induced model to generate all the complications found in MetS. To this end, we fed both WT and APP/PSN1 with a high-fat diet (HFD) in the presence of either ApN or resistin at a pre-symptomatic disease stage (4 months old) three times a week for 16 weeks (**Figure 1A**) (52). Body weights, calorie intake, and other critical parameters related to obesity and insulin resistance were measured during the 16-week feeding period. At the end of treatment, WT mice fed with HFD showed a significant increase in body weight and calorie intake compared to WT mice fed with a control diet (**Table 1**). However, no significant changes in body weights were observed between WT mice fed with HFD and those receiving the injections of ApN or resistin. Moreover, disturbances in several parameters related to systemic metabolic alteration, including changes in glucose, cholesterol, triglycerides, insulin, and HOMA were detected in all the groups fed with HFD except for those treated with ApN. Therefore, these results indicate that at least 3 of the 5 conditions present in metabolic syndrome were induced in the present study, confirming the induction of MetS in the HFD and HFD treated with resistin. Importantly, ApN was able to reverse all the aforementioned altered parameters in both the WT and APP/PSN1 animals fed with HFD. Next, we also compared glucose levels in CSF of WT and APP/PSN1 animals (**Table 2**). In both WT and transgenic animals, we observed a significant increase in glucose levels in the HFD group compared to corresponding controls. Interestingly, ApN administration to HFD animals caused a reduction in glucose concentration, while changes were observed after resistin, compared to HFD animals. Our results also indicated that WT and APP/PSN1 mice fed with HFD had significantly lower levels of ApN and increased levels of resistin in the CSF relative to controls (**Table 2**). Moreover, animals treated with either ApN or resistin showed the expected increase in the levels of the respective adipokines in both WT and APP/PSN1 mice fed with HFD.

**Table 1:** General parameters and blood measurements of WT and APP/PSN1 animals treated with HFD plus adiponectin or resistin.

	WT	WT+HFD	WT+HFD+ApN	WT+HFD+Resistin
Body weight (g)	31 ± 9	50 ± 8*	54 ± 7*	56 ± 8*
Calories intake (Kcal/day)	24 ± 3	38 ± 5*	41 ± 5*	43 ± 4**
Adiponectin (ug/mL)	15 ± 3	8 ± 2*	19 ± 3*^&^	8 ± 2*
Resistin (ng/mL)	7 ± 2	17 ± 2*	8 ± 2*	30 ± 5**^&^
Glucose (mg/dL)	91 ±19	155 ± 23*	93 ± 19^&^	177 ± 20**
Cholesterol (mg/dL)	132 ± 22	190 ± 21*	126 ± 20	200 ± 28*
Triglycerides (mg/dL)	85 ± 13	140 ± 21**	111 ± 18^&^	160 ± 19**
Insulin (mg/dL)	1 ± 0.1	3 ± 0.2**	1.3 ± 0.2^&^	3.5 ± 0.2**
HOMA (mg/dL)	0.2 ± 0.03	1.1 ± 0.1**	0.3 ± 0.1^&^	1.2 ± 0.1**
	APP/PSN1	APP/PSN1+HFD	APP/PSN1+HFD+ApN	APP/PSN1+HFD+Resistin
Body weight (g)	31 ± 8	56 ± 9*	50 ± 9*	58 ± 8*
Calories intake (Kcal/day)	21 ± 3	42 ± 6**	44 ± 7**	46 ± 6**
Adiponectin (ug/mL)	12 ± 2	6 ± 3*	20 ± 5*^&^	7 ± 1*
Resistin (ng/mL)	9 ± 2	26 ± 4**	25 ± 5**	45 ± 8**^&^
Glucose (mg/dL)	97 ± 21	173 ± 15**	105 ± 15^&^	170 ± 23**
Cholesterol (mg/dL)	123 ± 20	196 ± 22*	145 ± 22	231 ± 38**
Triglycerides (mg/dL)	81 ± 18	156 ± 23**	104 ± 25^&^	162 ± 25*
Insulin (mg/dL)	0.8 ± 0.1	3.4 ± 0.9**	1.2 ± 0.3^&^	3.7 ± 0.8**
HOMA (mg/dL)	0.1 ± 0.02	1.5 ± 0.1**	0.5 ± 0.1^&^	1.7 ± 0.2*

*p < 0.05 and **p < 0.01, compared with WT or APP/PSN1 control groups; ^&^p < 0.05, compared with WT or APP/PSN1 + HFD group.

**Table 2:** Adiponectin, resistin and glucose levels in the CSF from WT and APP/PSN1 animals treated with HFD plus adiponectin or resistin.

	WT	WT+HFD	WT+HFD+ApN	WT+HFD+Resistin
Adiponectin (ng/mL)	14.5 ± 2.1	3.3 ± 0.5**	10.8 ± 0.9&	3 ± 0.6**
Resistin (pg/mL)	9.6 ± 2.7	34.5 ± 3**	28 ± 2.8**	54 ± 4*^&^
Glucose (mg/dl)	58 ± 3.6	93 ± 5***	64 ± 3.5^&&&^	96 ± 3***
	APP/PSN1	APP/PSN1+HFD	APP/PSN1+HFD+ApN	APP/PSN1+HFD+Resistin
Adiponectin (ng/mL)	12.6 ± 0.7	3.6 ± 0.4*	10 ± 0.4^&^	4 ± 0.8*
Resistin (pg/mL)	12.3 ± 1.5	24.2 ± 1.3*	21 ± 1.1*	40 ± 2.9*^&^
Glucose (mg/dl)	71 ± 3.5	107 ± 4.3***	67 ± 4.8^&&&^	100 ± 5.8**

*p < 0.05 and **p < 0.01, compared with WT or APP/PSN1 control groups; ^&^p < 0.05, compared with WT or APP/PSN1 + HFD group.

### ApN stimulates brain glucose uptake in the cortex and hippocampus of both WT and APP/PSN1 mice, whereas resistin decreases it

Once MetS was established, we examined whether this condition was able to alter glucose metabolism in WT and APP/PSN1 mice. To this end, we injected mice with radioactive glucose and measured glucose uptake in the cortex and hippocampus. Our results showed a significant drop in glucose uptake in both cortex and hippocampus in WT and APP/PSN1 HFD-fed mice, relative to controls (**Figures 1B, C**). Importantly, ApN-treated mice recovered the uptake of glucose to levels similar to controls, while no effect was seen by resistin treatment. To take up glucose, the brain depends on several carriers, such as glucose transporters (Gluts) (53). Thus, we measured the mRNA levels of Glut1, which is abundant in the blood-brain barrier, Glut3, the canonical transporter in neurons and Glut4, which is expressed in several brain regions (54). We did not observe any differences in Glut1 expression in any of the different conditions (**Figure 1D**). However, HFD caused a significant decrease in the expression of Glut3 in the cortex and hippocampus of WT mice relative to controls, whereas in APP/PSN1 mice, Glut3 was only reduced in the hippocampus (**Figure 1E**). Moreover, the administration of ApN restored the expression of Glut3 to that of control levels in WT mice and only in the hippocampus of APP/PSN1 mice. Contrary to ApN, the administration of resistin, significantly lowered the expression of Glut3 in HFD-fed WT mice and the hippocampus of APP/PSN1 mice, relative to control, reaching similar levels to the HFD condition. Strikingly, no differences in Glut3 expression levels were observed after any of the treatments on the cortex of APP/PSN1 animals. Similar to Glut1, Glut4 expression levels were not altered in any WT groups (**Figure 1F**). However, its expression significantly decreased in the hippocampus, but not in the cortex, of APP/PSN1 HFD-fed mice. Interestingly, ApN treatment was able to recover Glut4 expression levels in the hippocampus, while resistin caused a reduction in both brain regions. Although Glut4 was initially described for its role in glucose uptake in skeletal muscle, recent studies have also highlighted its presence and function in the brain (55, 56). In fact, Glut4 is predominantly expressed by neurons in various brain regions, including the hippocampus and cortex (57). Similar to its function in peripheral tissue, cerebral Glut4 activity is influenced by insulin, but other factors, such as neuronal activity, can also regulate its activity (54, 57–59). While its exact physiological role is still under debate, Glut4 is believed to be involved in memory regulation and providing energy support during sustained synaptic function (54, 60). Interestingly, McNay’s group observed impaired spatial memory and reduced Glut4 translocation in rats after the administration of Aβ_42_ oligomers (61).

### ApN administration restores glucose metabolism in hippocampal slices

**Figure 2:**
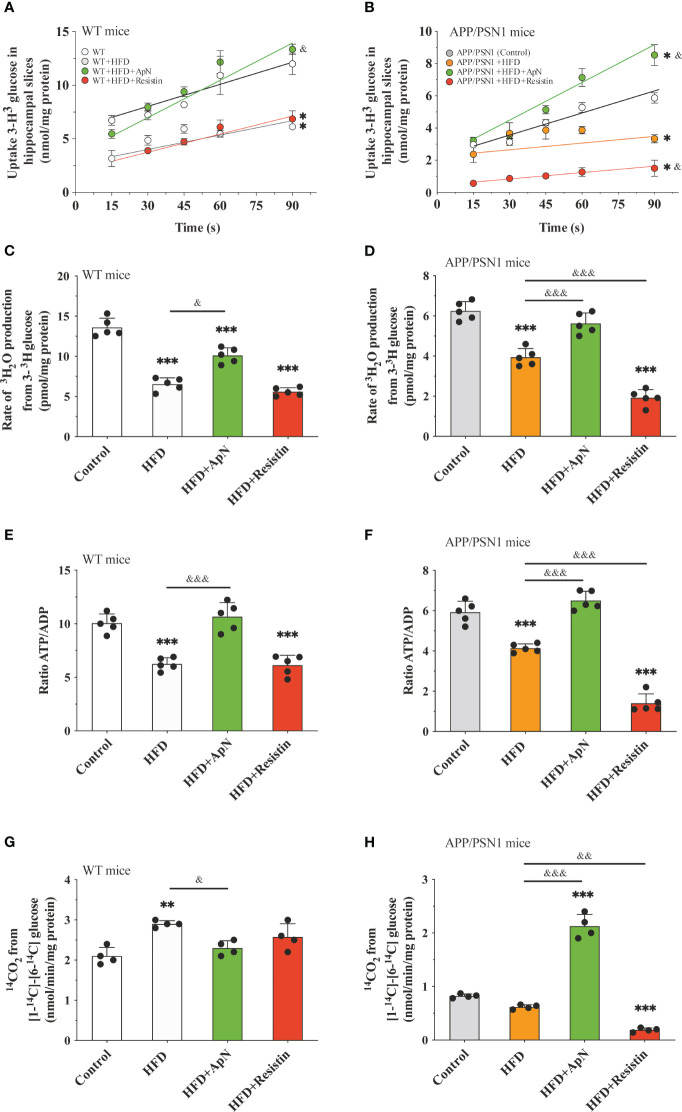
Glucose metabolism is restored after ApN treatment in hippocampal slices Hippocampal slices were obtained from WT or APP/PSN1 mice treated with either ApN or resistin and the **(A, B)** uptake of glucose, **(C, D)** glycolytic flux, **(E, F)** ATP/ADP ratio and **(G, H)** pentose phosphate flux (PPP) were measured. Means ± SEM are shown (n = 4-5 mice per condition in 3 technical replicates). Statistical significance was determined by either one-way ANOVA **(C-H)** or two-way ANOVA **(A)**, followed by Bonferroni’s multiple comparison posthoc test. *p < 0.05, **p < 0.01 and ***p < 0.001, compared with control group; ^&^p < 0.05 and ^&&^p < 0.01, ^&&&^p < 0.001 compared with specified group.

Once glucose is transported into the cell, it can be converted into glucose-6-phosphate, which in turn is metabolized by both the glycolytic and the pentose phosphate pathway (PPP) (62). To evaluate whether the observed alterations in glucose uptake could lead to disturbances in glucose metabolism, next, we measured glucose uptake, glycolytic and PPP fluxes, and the ATP/ADP ratio in hippocampal slices. Similar to our *in vivo* results, we observed that HFD caused a drop in glucose uptake in WT animals, which was reversed by ApN treatment, while resistin treatment did not cause a significant change compared to the control (**Figure 2A**). In the transgenic group, HFD also resulted in reduced glucose uptake, a condition that was further exacerbated by resistin and recovered by ApN (**Figure 2B**). As expected, our results showed that the rate of glycolysis in both WT and APP/PSN1 animals fed with HFD significantly decreased, while ApN and resistin treatment increased and reduced the glycolytic rate as compared to HFD, respectively (**Figures 2C, D**). Next, to determine whether the alterations in glucose utilization by glycolysis have an effect on ATP synthesis and to discriminate between ATP release or synthesis, we also measured ATP/ADP ratio. We found that HFD significantly reduced the ATP/ADP ratio in glycolysis in both WT and APP/PSN1, while ApN increased it (**Figures 2E, F**). Interestingly, resistin treatment in APP/PSN1 HFD-fed animals had a more pronounced effect on the ATP/ADP ratio as compared to WT mice. On the other hand, no differences were observed in the rate of glucose oxidized through the PPP in WT mice in any of the treatments (**Figures 2G, H**). However, the rate of glucose utilization through PPP in ApN treated APP/PSN1 mice was increased approximately 2.5-fold relative to HDF control, whereas resistin treatment further decreased PPP relative to controls.

### MetS-induced cognitive failure is recovered by ApN and further worsened by resistin treatment

**Figure 3:**
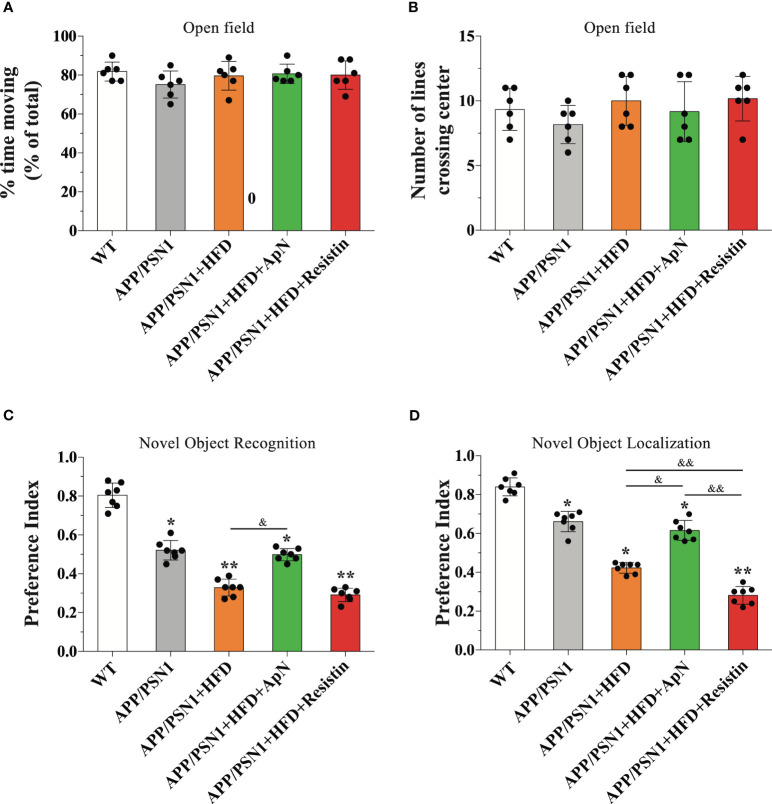
ApN recovered and resistin further deteriorated cognitive failure induced by MetS. WT and APP/PSN1 mice fed with control or HFD in the presence of either ApN or resistin were subjected to cognitive tests, including open field **(A)** percentage of time moving and **(B)** the number of crossing lines, **(C)** novel object localization (NOL) and **(D)** novel object recognition (NOR). Means ± SEM are shown (n = 6-7 mice per condition in 3 technical replicates). Statistical significance was determined by one-way ANOVA, followed by Bonferroni’s multiple comparison posthoc test. *p < 0.05 and **p < 0.01, compared with control group; ^&^p < 0.05 and ^&&^p < 0.01, compared with specified group.

To evaluate whether HFD and ApN or resistin treatment impact behavioral performance, we subjected mice to an open field test to assess for anxiety and general behavioral activity. No changes were observed among the treatments in WT or APP/PSN1 mice (**Figures 3A, B**). Next, to evaluate spatial recognition and to examine the impact of short-term memory we performed novel object localization (NOL) and novel object recognition (NOR) tests, respectively. Our results showed that inducing MetS generated a cognitive failure in APP/PSN1 mice, as compared with transgenic mice fed with control diet (**Figures 3C, D**). More importantly, ApN was able to revert the cognitive impairment in both NOL and NOR. However, resistin-treated APP/PSN1 animals showed no differences in the preference index in NOR, but further worsened the impairment in NOL, as compared to untreated animals, respectively.

### ApN restores the Aβ_42_/Aβ_40_ ratio and reduces the levels of Aβ aggregates in both hippocampus and cortex in MetS-induced APP/PSN1 animals

**Figure 4:**
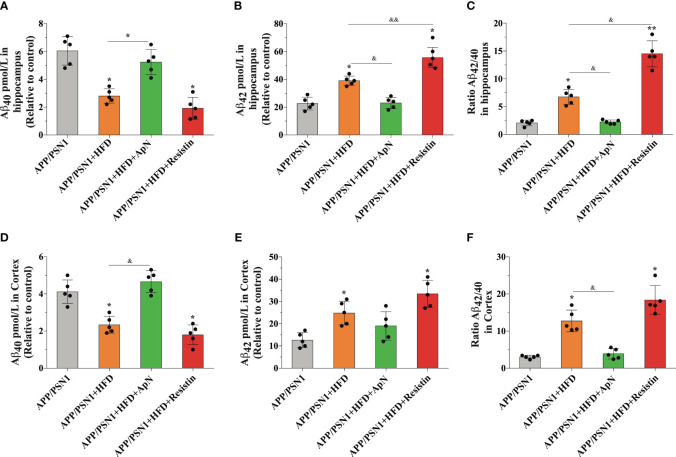
ApN restores Aβ_42_/Aβ_40_ ratio in the hippocampus of MetS-induced APP/PSN1 mice. **(A, B)** the levels of Aβ_40_ and Aβ_42_ were measured in the hippocampus of APP/PSN1 mice fed with control or HFD in the presence of either ApN or resistin and **(C)** the Aβ_42_/Aβ_40_ ratio was calculated accordingly. **(D, E)** the levels of Aβ_40_ and Aβ_42_ were measured in the hippocampus of APP/PSN1 mice fed with control or HFD in the presence of either ApN or resistin and **(F)** the Aβ_42_/Aβ_40_ ratio was calculated accordingly. Means ± SEM are shown (n = 5 mice per condition in 3 technical replicates). Statistical significance was determined by one-way ANOVA, followed by Bonferroni’s multiple comparison posthoc test. *p < 0.05 and **p < 0.01, compared with control group; ^&^p < 0.05 and ^&&^p < 0.01, compared with specified group.

**Figure 5:**
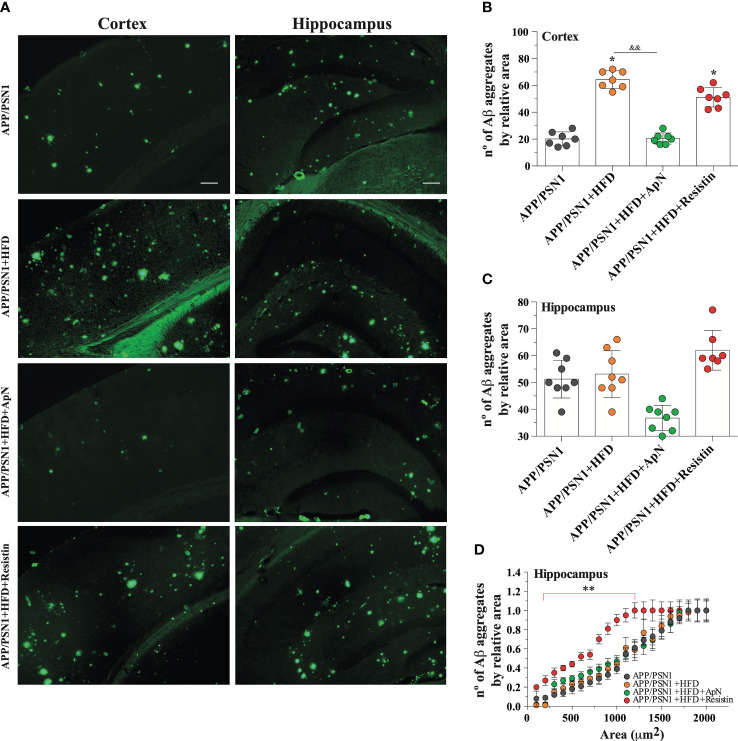
ApN reduces Aβ deposition in HFD-fed APP/PSN1 mice. **(A)** Representative images of thioflavin S staining in the cortex or hippocampus of APP/PSN1 mice fed with either control diet or HFD in the presence of ApN or resistin. Scale bar: 200 μm. **(B, C)** Quantification of the number of amyloid plaques in the aforementioned conditions. **(D)** Quantification of the number of amyloid plaques by sectional area relative to plaque size (μm) in cortical or hippocampal slices in the aforementioned conditions. Means ± SEM are shown (n = 7-8 mice per condition in 3 technical replicates). Statistical significance was determined by one-way ANOVA, followed by Bonferroni’s multiple comparison *post-hoc* test. *p < 0.05 and **p < 0.01, compared with control group; ^&&^p < 0.01, compared with specified group.

Aβ deposition is a common hallmark in AD and it is associated with brain dysfunction, which leads to cognitive impairment. Therefore, we next measured Aβ_40_ and Aβ_42,_ two of the most abundant Aβ species in amyloid plaques, with the latter being more prone to aggregation. Inducing MetS produced a significant decrease in Aβ_40_ levels and an increase in Aβ_42_ in the hippocampus of APP/PSN1 animals, as compared to controls (**Figures 4A, B**). Importantly, ApN administration reverted this effect to levels similar to controls, whereas resistin further aggravated it. As for the Aβ_42_/Aβ_40_ ratio, the levels were exacerbated by HFD, with ApN restoring it and resistin further increasing it (**Figure 4C**). Similar results as the hippocampus were obtained for the cortex, however, the effect seems to be less pronounced (**Figures 4D–F**). To further analyze the effect of ApN and resistin in Aβ deposition, we next measured the amount of Aβ aggregates in both the cortex and hippocampus. Our results showed a significant increase in cortical Aβ aggregates in MetS-induced and resistin-treated APP/PSN1 animals compared to controls, while ApN treatment caused a significant reduction in cortical Aβ deposition (**Figures 5A, B**). On the other hand, we observed similar levels of Aβ aggregates between control APP/PSN1 group and those fed with HFD in the hippocampus (**Figures 5A, C**). Moreover, ApN decreased the levels of Aβ aggregates, relative to HFD-fed APP/PSN1 animals, while no changes were observed after resistin treatment. Interestingly, quantitative analysis of the area covered by these aggregates showed only differences in the resistin-treated group, with a significantly lower size (**Figure 5D**).

### Resistin increases GFAP and 4-HNE levels in the hippocampus of MetS-induced mice

**Figure 6:**
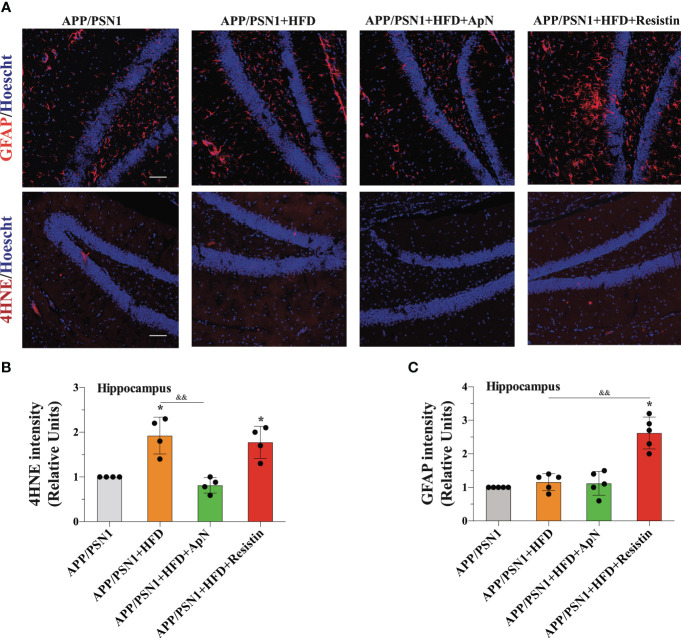
GFAP and 4-HNE levels are increased after resistin treatment in the hippocampus of MetS-induced APP/PSN1 mice. **(A)** Representative images of GFAP or 4-hydroxynonenal (4-HNE) from hippocampal slices of APP/PSN1 brains fed with either control diet or HFD in the presence of ApN or resistin. **(B)** Quantification of GFAP levels in hippocampal slices. **(C)** Quantification of 4-HNE levels in hippocampal slices. Means ± SEM are shown (n = 4-5 mice per condition in 3 technical replicates). Statistical significance was determined by one-way ANOVA, followed by Bonferroni’s multiple comparison posthoc test. *p < 0.05, compared with control group; ^&&^p < 0.01, compared with specified group. Scale bar: 150 μm.

Finally, to determine whether MetS could be generating a neurotoxic effect through inflammation, we analyzed the glial marker GFAP and 4-hydroxynonenal (4-HNE), a marker of lipid peroxidation in the hippocampus of APP/PSN1 mice. Our analysis showed a significant increase in GFAP only in the animals treated with resistin, while no changes were observed in the other groups (**Figures 6A, C**). Interestingly, 4-HNE was increased in MetS-induced animals, an effect that was reverted by ApN and further exacerbated by resistin treatment (**Figures 6A, B**).

## Discussion

**Figure 7:**
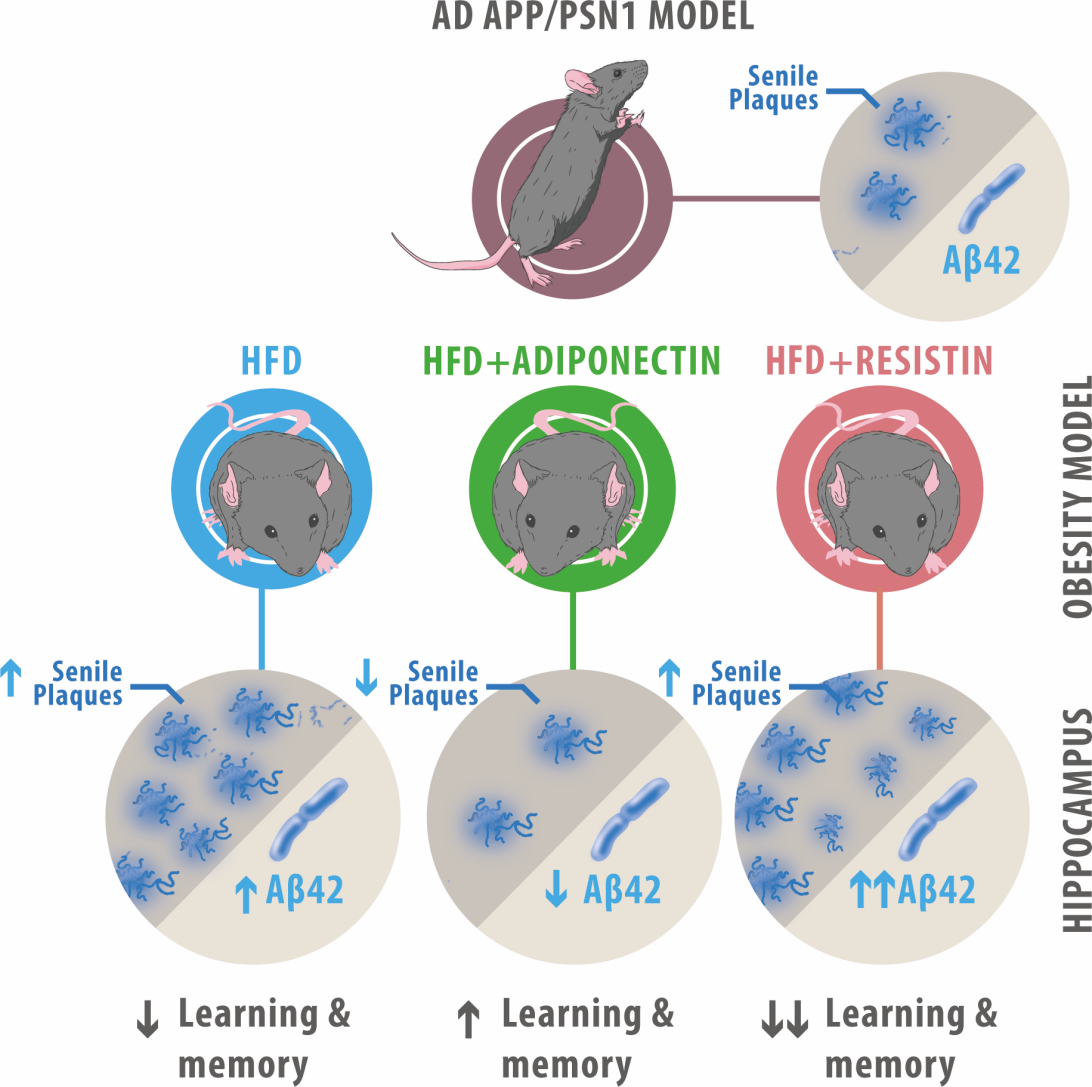
Summary of ApN and resistin effects on APP/PSN1 animals fed with HFD. HFD-fed APP/PSN1 mice showed an increased number of senile plaques and Aβ_42_ levels, along with a decrease in cognitive performance, effects that were further worsened by resistin treatment. Conversely, administration of ApN to HFD-fed APP/PSN1 mice resulted in improved cognitive performance and reduced levels of senile plaques and Aβ_42_ levels.

The presented work demonstrates that ApN and resistin, adipokines known to be altered during obesity, affect metabolic and cognitive functions. ApN treatment caused an overall metabolic and cognitive improvement while reducing the number of Aβ plaques in AD transgenic models fed with HFD (**Figure 7**). In contrast, resistin caused detrimental effects, including a decrease in glucose uptake, an increase in the expression of oxidative markers and the levels of Aβ_42_, together with higher Aβ deposition, alterations associated with AD pathology.

The establishment of the MetS condition in both WT and transgenic APP/PSN1 is consistent with other studies, where the global metabolism was also altered. The importance of establishing MetS is due to the clinical impact that this represents since MetS usually precedes the more severe pathologies like type 2 diabetes. Accumulating evidence has also shown MetS to play a crucial role in the brain and increase resilience to neurological disorders. Indeed, induction of MetS was shown to increase the incidence of cognitive disorders in aging and AD, even in young individuals (63). In fact, epidemiological studies have suggested a strong positive correlation between MetS and AD (64, 65). Our findings are consistent with these observations and demonstrate that MetS decreases cognitive function, specifically hippocampal-dependent learning and memory (as judged by normal object recognition and localization).

It is worth noting that AD mouse models exhibit an altered glucose metabolism under basal conditions and that HFD has been shown to impair cognitive performance also in wild-type mice (66–70). However, HFD appears to exacerbate the cognitive decline in different AD mice models and several reports show a strong association between MetS and AD in humans (71, 72, 73, 64, 65, 74–77). Among the common pathological features between AD and MetS is insulin resistance, which has been shown to promote the clearance of Aβ and affect Aβ aggregation (78, 79). Interestingly, insulin has also been suggested to regulate memory and numerous studies have demonstrated alterations in insulin signaling in AD patients (80–82). A different overlapping mechanism between MetS and AD is glucose metabolism. Indeed, a vast body of evidence has shown impairments in glucose metabolism in the brains of AD patients (4, 83).

Cognitive decline in HFD-fed animals was also associated with metabolic alterations. We observed an increase in glucose uptake in the hippocampus and cortex after ApN treatment, along with an increase in Glut3 and Glut4 expression, whereas resistin treatment had the opposite effects. The increase in the levels of Glut3 and Glut4 caused by ApN in the HFD model suggests that adipokines can modulate the expression and/or translocation of these transporters to the membrane to increase glycolysis. However, we did not see changes in the expression of Glut1 after ApN or resistin treatment, which suggests that Glut3 and Glut4 may be responsible for the overall shift in glucose uptake. Alternatively, ApN or resistin might not have a direct effect on glucose transporters but rather an intracellular effect through the modulation of AMPK or hexokinase, among others. A dysfunction in glucose uptake has also been strongly associated with AD pathogenesis, and it is manifested several years before patients show clinical symptoms (84, 85). Moreover, numerous studies suggest that promoting glucose uptake or increasing Glut expression can improve cognitive performance and protect neurons against Aβ toxicity (52, 86, 87). Our results suggest that the presence of ApN increases glucose uptake, glycolysis, and ATP/ADP ratio in AD transgenic mice, whereas resistin treatment reduces it. Interestingly, ApN has been described as an insulin sensitizer that improves insulin sensibility via activation of AMPK, a downstream effector of the ApN receptor, promoting Glut trafficking and translocation to the membrane (88–90). Indeed, it has been shown that ApN, similar to insulin, is able to remodel the cytoskeleton, which results in Glut4 translocation into the membrane (91, 92). Conversely, the absence of ApN results in AMPK inactivation, which increases phosphorylation at serine residues of the insulin receptor substrate-1, one of the primary effectors of the insulin growth factor receptor, decreasing insulin sensitivity (93). On the other hand, resistin has been shown to reduce glucose sensitivity and uptake, possibly through modulating the Akt pathway (94, 95). In this regard, resistin has been implied as a risk factor for dementia since it antagonizes insulin action and promotes inflammation in obesity (96). Interestingly, recent studies with AD patients described elevated levels of resistin compared to controls, suggesting a possible association between resistin levels and higher risks of dementia, however, these results are controversial (97–99).

Consistent with earlier studies, ApN treatment results in a significant reduction of Aβ_1-42_ levels, while ApN deficiency accelerates Aβ pathology (93, 100–102, 103). Hahm and coworkers proposed that HFD consumption causes metabolic perturbations and ApN deficiencies that suppress ApN receptor-mediated AMP-activated protein kinase (AMPK) phosphorylation. In turn, this alteration elevates NF-kb and JNK levels leading to increased Beta-secretase 1 (BACE1) protein levels and Aβ production (93). Importantly, ApN deficiency promotes tau phosphorylation in the hippocampus and frontal cortex, which is known to increase amyloid plaque burden and exacerbate AD pathology (104). Furthermore, high ApN concentrations have been shown to protect human neuroblastoma cells (SH-SY5Y) against Aβ_1-42_ induced neurotoxicity, which is achieved via stimulation of the glucose metabolism (31, 105). Unlike ApN, resistin caused smaller Aβ plaque size but increased Aβ_1-42_ peptides. This difference between adipokines could arise because resistin treatment resulted in a larger number of Aβ_1-42_ peptides in the brain, creating more aggregation points. Thus, Aβ_1-42_ peptides divide and accumulate among a larger number of seeds, resulting in smaller plaques. This result contrasts with an earlier study from Jie et al., who did not observe Aβ_1-40_ or Aβ_1-42_ changes after resistin treatment in mouse cell lines that overproduce Aβ (106). The difference may be due to the effects of resistin in cultured cell lines versus its effects in the mouse brain.

Thus, further studies are needed to elucidate the exact molecular function and mechanism of action of resistin.

Among the limitation of our study is the age of the animals utilized for assessing the impact of the adipokines. Notably, a recent study reported that the effect of ApN varies depending on age. While ApN demonstrates a beneficial, neuroprotective, and anti-neurodegenerative in reproductive stages, it exhibits a detrimental effect in human aging, a phenomenon known as the ApN paradox (107, 108). Moreover, APP and PS1 transgenes have been shown to increase mRNA expression of adiponectin receptors (109). It is also worth noting that AD is a multifactorial disease involving interactions between genes, environmental factors, and lifestyle factors. Animal models may simplify these complexities, limiting their ability to represent the disease fully and to project the results to humans.

Another characteristic hallmark of AD is the abnormal activation of astrocytes, which become reactive in response to pro-inflammatory signals and excessive reactive oxygen species, rendering the brain more susceptible to neuronal damage (110, 111). Interestingly, astrogliosis and oxidative stress have also been observed in obese individuals, suggesting a possible connection between these two conditions (112). In our study, resistin treatment caused an increase in the oxidative marker 4HNE, together with a rise in GFAP levels. These observations are consistent with previous results showing that resistin levels positively correlate with oxidative stress (113). A resistin-mediated increase in GFAP levels has also been reported, however, these observations remain controversial (114, 115). Contrarily, we observed a reduction in 4HNE after the administration of ApN, suggesting that ApN protects against oxidative damage (103, 116, 117).

In conclusion, the data presented in this study suggest and provide additional support for the notion that obesity-associated changes affect both cognition and metabolism in both wild-type and AD models. Importantly, ApN was able to rescue phenotypes caused by HFD, while resistin worsens the clinical condition. Our study contributes to understanding the mechanisms by which obesity could aggravate AD progression and may offer a preventive and potential therapeutic avenue to restore AD metabolism. Nevertheless, further work is needed to clarify how ApN and resistin play a beneficial role in AD.

## Data availability statement

The raw data supporting the conclusions of this article will be made available by the authors, without undue reservation.

## Ethics statement

The animal study was approved by The Bioethical and Biosafety Committee of the Faculty of Biological Sciences of the Pontificia Universidad Católica de Chile. The study was conducted in accordance with the local legislation and institutional requirements.

## Author contributions

Conceived and designed the experiments: PC and NI. Performed the experiments: PC, CG, CM-O, JG, GW, and PS. Analyzed the data: PC and NI. Contributed reagents/materials/analysis tools: PC and NI. Wrote the manuscript: PC, CG, PS, and NI. All authors contributed to the article and approved the submitted version.
